# Bayesian Compressive Sensing Based Optimized Node Selection Scheme in Underwater Sensor Networks

**DOI:** 10.3390/s18082568

**Published:** 2018-08-06

**Authors:** Ruisong Wang, Gongliang Liu, Wenjing Kang, Bo Li, Ruofei Ma, Chunsheng Zhu

**Affiliations:** 1 Harbin Institute of Technology, Weihai 264209, China; mathwrs@163.com (R.W.); liugl@hit.edu.cn (G.L.); libo1983@hit.edu.cn (B.L.); maruofei@hit.edu.cn (R.M.); 2 Department of Electrical and Computer Engineering, The University of British Columbia, Vancouver, BC V6T 1Z4, Canada; chunsheng.tom.zhu@gmail.com

**Keywords:** Bayesian estimation, compressed sensing, network lifetime, underwater sensor network

## Abstract

Information acquisition in underwater sensor networks is usually limited by energy and bandwidth. Fortunately, the received signal can be represented sparsely on some basis. Therefore, a compressed sensing method can be used to collect the information by selecting a subset of the total sensor nodes. The conventional compressed sensing scheme is to select some sensor nodes randomly. The network lifetime and the correlation of sensor nodes are not considered. Therefore, it is significant to adjust the sensor node selection scheme according to these factors for the superior performance. In this paper, an optimized sensor node selection scheme is given based on Bayesian estimation theory. The advantage of Bayesian estimation is to give the closed-form expression of posterior density function and error covariance matrix. The proposed optimization problem first aims at minimizing the mean square error (MSE) of Bayesian estimation based on a given error covariance matrix. Then, the non-convex optimization problem is transformed as a convex semidefinite programming problem by relaxing the constraints. Finally, the residual energy of each sensor node is taken into account as a constraint in the optimization problem. Simulation results demonstrate that the proposed scheme has better performance than a conventional compressed sensing scheme.

## 1. Introduction

Compared to the traditional sensor networks [1,2], the underwater sensor network is more restricted to the resource. The study of underwater sensor networks has become a hot topic and has attracted more attention in recent years [3,4,5,6]. For example, routing protocols and secure communication for underwater sensor networks are investigated in [7,8], respectively. However, none of them focus on the full use of node energy resources. However, the energy of each sensor node is fixed and limited in underwater sensor networks. A traditional information acquisition scheme needs to collect the information of all the sensor nodes so that it costs too much energy. Hence, some works are provided for decreasing the price of sensor networks [9,10]. In addition, it is difficult for the sink node to distinguish the signals from different sensor nodes. An efficient and affordable scheme needs to be proposed for superior performance. A compressed sensing method can be used to solve it as long as the original signal is sparse [11]. In the underwater sensor network, many kinds of signals received by sink nodes can be represented sparsely on some basis such as temperature and salinity. Therefore, we can collect a part of the total sensor nodes and then reconstruct the original signal. The compressed sensing scheme can not only decrease the cost but also improve the efficiency. The state of network and the correlation of nodes are not considered due to the randomness of node selection in a conventional compressed sensing scheme. Hence, the performance of the compressed sensing scheme can be further improved by considering them.

### 1.1. Related Works and Motivation

Compressed sensing, which has been studied by many scholars [12,13,14,15], is a vital technique for decreasing the samples in the information acquisition. For example, the authors in [16] applied a compressed sensing method to multipath channel estimation. In [17], the authors designed an optimized compressed sensing matrix and applied it to the sparse channel estimation. In [18], the directions of arrival estimation were studied by a compressed sensing method and it is robust and very accurate. Compressed sensing theory is also used to develop the practical incoherent undersampling schemes for rapid MR imaging [19]. By using a compressed sensing method, a semi-analytic scheme is developed to solve the iterative clipping noise recovery in [20]. By taking advantage of the compressed sensing method, the authors in [21] addressed nonlinear inverse scattering problems. Furthermore, examples with experimental data also showed the effectiveness of the compressed sensing method in position and shape reconstruction [22]. In addition, compressed sensing theory can be used to cancer imaging about the induced magnetic anomaly [23]. Therefore, it is it is of significance to develop the application of the compressed sensing technique. In recent years, Bayesian compressive sensing has been widely investigated due to its advantages of giving posterior density function and error covariance matrix. For instance, a Bayesian compressive sensing method is used to estimate complex-valued targets in wireless localization systems [24]. Thermal imaging is studied with an application of Bayesian compressive sensing in [25]. In [26], the authors provided an estimation for direction of arrival based on Bayesian compressive sensing and gave a Kalman filter to analyze the signals. A Bayesian compressive sensing framework was used to develop the channel state information (CSI) acquisition scheme by considering the parametric sparsity and channel model in [27]. In [28], the authors studied Bayesian framework and provided some useful results and algorithms. In [29], ultra-wideband channel estimation is investigated by making use of Bayesian estimation theory. The authors also analyzed the performance and the application situations of this scheme.

Although there are some works about the application of compressed sensing in underwater sensor networks [30,31,32,33,34], few studies focused on the effect of the sensor node selection scheme. For underwater sensor networks, the energy is strictly limited because the underwater sensor node can not be charged again. It is significant to consider the sustainability of the network when the sink node determines the selection set of sensor nodes. In addition, to obtain more accurate reconstruction results, the performance of reconstruction is worthy of studying. Motivated by the analysis above, we aim at providing a novel sensor node selection scheme for improving the reconstruction performance and the network lifetime. By making full use of the advantage of Bayesian theory, the mean square error (MSE) of estimation can be utilized to optimize the sensor node selection scheme.

### 1.2. Contributions

The paper considers an underwater sensor network where there is a sink node and the sensor nodes transmit the information to it directly. Suppose that the sink node knows the location of each sensor node. Combining Bayesian theory and convex optimization theory, we investigated an optimized sensor node selection scheme based on compressed sensing in underwater sensor networks. The proposed scheme can be obtained by solving an optimization problem. Both the network lifetime and reconstruction accuracy performance are considered in the optimization problem. In this paper, our main contributions can be summarized as follows:

- The paper focuses on the improvement of performance of a compressed sensing scheme. First, a Bayesian estimation theory is provided for the signal reconstruction. Then, based on Bayesian estimation theory, the closed-form expression of posterior density function and error covariance matrix are given. By a maximum posteriori estimation, the noise variance can be obtained by updating iteratively.
- The sensor node selection scheme is transformed as a sensing matrix design problem. By using the error covariance matrix of Bayesian estimation and regarding the sensing matrix as a variable, the sensing matrix design problem can be treated as an optimization problem of minimizing the MSE. Because the proposed optimization problem is non-convex, the optimal solution is difficult to obtain. By relaxing the integer constraint as a continuous constraint, the proposed optimization problem becomes a convex semidefinite programming problem that can be solved efficiently.
- The sustainability of networks is considered in the proposed optimization problem. To prolong the network lifetime, the scheme aims at selecting the sensor nodes holding more residual energy. This idea is transformed into a constraint in the optimization problem.

The proposed sensor node selection scheme is evaluated by simulation results. Simulation results demonstrate that the optimized node selection scheme has superior performance compared to the traditional one.

### 1.3. Paper Organization

The rest of the paper is organized as follows. The compressed sensing theory, Bayesian method, and the underwater channel model are introduced in Section 2. We propose the optimization problem about the MSE and the energy limit in Section 3. Section 4 provides the simulation results to show the superior performance of the proposed scheme. A conclusion of the paper is given in Section 5.

### 1.4. Notation

The following notations are used in the rest of the paper. Let the bold fonts denote vectors or matrices. The transpose operations are denoted by ${\left(\cdot \right)}^{T}$, respectively. Let ${∥\cdot ∥}_{2}$ denote the (Euclidean) vector norms. If there are no special instructions, the inequality between two vectors refers to the element of the vectors. The inverse of matrix is denoted as ${\left(\cdot \right)}^{-1}$. $I_{N}$ denotes the identity matrix with the dimension N×N.

## 2. System Model and Problem Formulation

### 2.1. System Model

The paper considers an underwater sensor network where a sink node is located in the center of a circular region and *N* sensor nodes are uniformly distributed in this range. The radius is *r*. Each sensor collects information such as temperature, salinity, and so on and then transmits them to a sink node. In the traditional information acquisition scheme, the sink node collects the information of all the sensor nodes that consume too much energy. To overcome this difficulty, a compressed sensing method can be applied to reduce the number of measurements. Suppose that the total signal $x={\left[x\left(1\right),x\left(2\right),\ldots ,x\left(N\right)\right]}^{T},$ where i=1,2,…,N can be expressed as

(1) $$x=\sum_{i=1}^{N}s\left(i\right)\Psi \left(i\right)=\Psi s,\;$$

where Ψ=[Ψ(1),Ψ(2),…,Ψ(N)] is a basis matrix and s is a sparse vector that there are K≪N nonzero elements and other elements are zero. The *K* is called the sparsity of vector s. The vector x is called compressible if the conditions above are satisfied.

According to the compressed sensing theory, the sink node can select M≪N measurements to reconstruct the original signal only if it is compressible in the basis Ψ. Let $\Phi \in R^{M\times N}$ denote the sensor node selection matrix which is independent from Ψ. The structure of node selection matrix Φ can be represented as follows:

(2) $$\left\{\begin{array}{c} \sum_{i=1}^{M}\Phi_{ij}\leq 1,j=1,2,\ldots ,N,\;\\ \sum_{j=1}^{N}\Phi_{ij}=1,i=1,2,\ldots ,M,\\ \Phi_{ij}\in 0,1,i=1,2,\ldots ,M,j=1,2,\ldots ,N. \end{array}\right.$$

If the *i*th element in the *j*th row of node selection matrix Φ is 1, it means that the *i*th node is selected. Then, the measurement vector can be obtained

(3) y=ΦΨs+n=As+n,

where A=ΦΨ is the projection matrix. The vector n is independent identically distributed (i.i.d) additive white Gaussian noise with mean zero and variance $\sigma^{2}$:

(4) $$p\left(n\right)=\prod_{i=1}^{N}N\left(n_{i}|0,\sigma^{2}\right).\;$$

The paper aims at reconstructing the sparse vector s instead of reconstructing the original signal vector x directly. From Equation (3), there are *N* variables and M≪N equalities. It is impossible to obtain the unique solution. However, notice that the vector s is sparse and has K≪N nonzero elements, and it makes the reconstruction possible if the projection matrix A meets the restricted isometry property (RIP). The reconstructed solution $\hat{s}$ can be obtained from the optimization problem as follows:

(5) $$\begin{array}{c} \begin{array}{c} {\arg\min∥s∥}_{0},\\ {s.t.\;∥y-As∥}_{2}\leq \varepsilon . \end{array} \end{array}$$

However, the optimization problem above is non-deterministic polynomial (NP)-hard due to the non-convexity of the objective function. To relax the original problem as a convex problem, a convex ${∥\cdot ∥}_{1}$ is usually used to replace the ${∥\cdot ∥}_{0}$. Then, the transformed problem can be represented as follows:

(6) $$\begin{array}{c} \begin{array}{c} {\arg\min∥s∥}_{1},\\ {s.t.\;∥y-As∥}_{2}\leq \varepsilon . \end{array} \end{array}$$

The problem above can be solved effectively by the CVX tool cabinet. The original signal $\hat{x}=\Psi \hat{s}$ can be recovered by using the solution $\hat{s}$. In the underwater information sampling, the signal is usually sparse due to the spatial correlation. For example, the temperature signal is sparse in the frequency domain, i.e., it can be expressed sparsely by Fourier basis. Therefore, it is significant to apply a compressed sensing technique for underwater data sampling.

### 2.2. Bayesian Compressive Sensing

In this subsection, the sparse vector s is reconstructed by measurement y according to Bayesian estimation. Compared with the traditional compressive sensing reconstruction algorithm, the advantage of Bayesian compressive sensing is that the noise variance can also be estimated. In addition, the posterior probability density function can be given through some prior information. The main idea of Bayesian estimation is taking full advantage of reasonably assumed prior information and the prior information including the distribution of noise and the measurement y. Firstly, the Gaussian likelihood model of the measurement y can be written as follows [28]:

(7) $$p\left(y|s,\sigma^{2}\right)={\left(2\pi \sigma^{2}\right)}^{-\frac{M}{2}}e^{-\frac{{∥y-As∥}_{2}^{2}}{2\sigma^{2}}}.$$

The posterior of the sparse vector s needs to be given to estimate the final solution. According to the Bayes’ rule, the prior information of the sparse vector s needs to be known. In general, Laplace prior is used in many works. However, it is not conjugate with the Gaussian likelihood model. The closed form solution of posterior distribution is difficult to obtain. Hence, a hierarchical prior is used in this paper instead of using it. Suppose that the element of the sparse vector s is an independent Gaussian distribution with zero mean and variance $\alpha_{i}^{-1}$. Then, the distribution of the sparse vector s can be shown:

(8) $$\begin{array}{c} \begin{array}{cc} p(s|\alpha ) & =\prod_{i=1}^{N}N\left(s_{i}|0,\alpha_{i}^{-1}\right)\\ & =\prod_{i=1}^{N}{\left(2\pi \right)}^{-\frac{1}{2}}\alpha_{i}^{\frac{1}{2}}e^{-\frac{\alpha_{i}s_{i}^{2}}{2}}, \end{array} \end{array}$$

where $\alpha ={\left(\alpha_{1},\alpha_{2},\ldots ,\alpha_{N}\right)}^{T}$ is the hyperparameter.

Hierarchical priors about the hyperparameters α, $\sigma^{2}$ and the sparse vector s are given in Appendix A. It is conjugate with Gaussian distribution. The marginal likelihood function $p(y|\alpha ,\sigma^{2})$ can be computed by integrating $p\left(y|s,\sigma^{2}\right)p\left(s|\alpha \right)$

(9) $$\begin{array}{c} \begin{array}{cc} p(y|\alpha ,\sigma^{2}) & =\int p\left(y|s,\sigma^{2}\right)p\left(s|\alpha \right)ds\\ & ={\left(2\pi \right)}^{-\frac{M}{2}}{\left|C\right|}^{-\frac{1}{2}}e^{-\frac{y^{T}C^{-1}y}{2}}, \end{array} \end{array}$$

where $C=\sigma^{2}I+A\Lambda^{-1}A^{T}$ and Λ is a diagonal matrix of the hyperparameters α.

According to the assumption of the hierarchical prior and the Gaussian likelihood model, the closed form solution of the posterior distribution can be derived:

(10) $$\begin{array}{c} \begin{array}{cc} & p(s|\alpha ,\sigma^{2},y),\\ = & {\left(2\pi \right)}^{-\frac{N}{2}}{\left|\Sigma \right|}^{-\frac{1}{2}}e^{-\frac{{\left(s-\mu \right)}^{T}\Sigma^{-1}\left(s-\mu \right)}{2}}, \end{array} \end{array}$$

where the mean and covariance matrix are μ and Σ as follows:

(11) $$\begin{array}{c} \begin{array}{c} \mu =\sigma^{-2}\Sigma A^{T}y\\ \Sigma ={\left(\sigma^{-2}A^{T}A+\Lambda \right)}^{-1}. \end{array} \end{array}$$

It can be seen that μ and Σ are functions of the hyperparameters α and $\sigma^{2}$ which are unknown. It is necessary to estimate them to obtain the mean and variance of the sparse vector s. The hyperparameters’ estimation can be obtained by maximizing $p\left(y|\alpha ,\sigma^{2}\right)p\left(\alpha \right)p\left(\sigma^{-2}\right)$. Due to a uniform distribution assumption of α and $\sigma^{2}$, the hyperparameters’ estimation can be obtained by maximizing the marginal likelihood function $p(y|\alpha ,\sigma^{2})$ directly, or equivalently maximizing the logarithm of $p(y|\alpha :\sigma^{2})$.

(12) $$\begin{array}{c} \begin{array}{cc} L & =\log p(y|\alpha ,\sigma^{2})\\ & =-\frac{1}{2}(M\log\left(2\pi \right)+\log|C|+y^{T}C^{-1}y). \end{array} \end{array}$$

By taking the derivative of L about α and setting it as zero, the estimation of α can be given as follows:

(13) $$\begin{array}{c} \begin{array}{c} \alpha_{i}=\frac{1}{\mu_{i}^{2}+\Sigma_{ii}}, \end{array} \end{array}$$

where $\Sigma_{ii}$ is the *i*th element of the covariance matrix Σ. By using the same method, the estimation of $\sigma^{2}$ can be obtained as follows:

(14) $$\begin{array}{c} \begin{array}{c} \sigma^{2}=\frac{{∥y-A\mu ∥}_{2}^{2}}{M-N+\sum_{i=1}^{N}\alpha_{i}\Sigma_{ii}}. \end{array} \end{array}$$

The derivations of Equations (13) and (14) are similar to [35]. It can be seen that the hyperparameters α and $\sigma^{2}$ are functions of the mean μ and covariance matrix Σ. The expectation maximization (EM) algorithm shown as Algorithm 1 can be used to update them iteratively. The iteration is terminated when there is a convergency value. Finally, the mean μ is regarded as the estimation of the sparse vector s. The original signal x can be reconstructed by using Ψμ.

**Algorithm 1** Expectation maximization algorithm for Bayesian estimation [35].
1:Initialize $\alpha_{i}\left[0\right],\forall i=1,2,\ldots ,N$ and $\sigma^{2}\left[0\right]$, set tolerance as ε and maximum number of iterations as *K*.2:
**for** k=1:K **do**
3: Calculate the mean μ[k] and covariance matrix Σ[k].4: Update the hyperparameters $\alpha_{i}\left[k\right],\forall i=1,2,\ldots ,N$ and $\sigma^{2}\left[k\right]$ according to the results of step 3.5: **if** $|\alpha_{i}\left[k\right]-\alpha_{i}\left[k-1\right]|<\varepsilon$ **then**6:  Break.7: **end if**8:
**end for**
9:Output the final mean μ[k] and covariance matrix Σ[k] and reconstruct the signal as Ψμ.


### 2.3. Underwater Channel Model

In the underwater environment, the signal transmission is usually achieved by acoustic transmission, which has characteristics of low speed and has long delay. Due to the reflection, refraction, and current, the pathloss in underwater transmission is large. The pathloss mode can be given as follows:

(15) $$\begin{array}{c} \begin{array}{c} A\left(d_{s},f\right)=d_{s}^{\tau }\phi {\left(f\right)}^{d_{s}}, \end{array} \end{array}$$

where $d_{s}$ is the distance between the sensor node and sink node, *f* is the carrier frequency, and τ is a constant which is usually set as 1.5.

The term ϕ(f) is called as absorption coefficient and given as follows:

(16) $$\begin{array}{c} \begin{array}{c} \phi \left(f\right)=10^{\frac{\lambda (f)}{10}}. \end{array} \end{array}$$

The λ(f) can be estimated by Thorps formula as

(17) $$\begin{array}{c} \begin{array}{c} \lambda \left(f\right)=\frac{0.11f^{2}}{1+f^{2}}+\frac{44f^{2}}{4400+f^{2}}+\frac{2.75}{10^{4}}f^{2}+0.003. \end{array} \end{array}$$

Ideal power control is assumed in this paper. Then, suppose that the power required by the sink node is $P_{0}$, and the sensor nodes need to take the power as least $P_{0}/A\left(d,f\right)$ to transmit the signal. Based on the analysis above, the energy cost model can be provided as follows:

(18) $$\begin{array}{c} \begin{array}{c} E_{i}=\left\{\begin{array}{cc} E_{c}+E_{u}A^{-1}\left(d_{s},f\right), & \mathrm{if}\;\mathrm{the}\;\mathrm{node}\;\mathrm{is}\;\mathrm{selected},\\ E_{c}, & \mathrm{otherwise}. \end{array}\right.\;\forall i=1,2,\ldots ,N, \end{array} \end{array}$$

where $E_{c}$ denotes the circuit energy consumption and $E_{u}$ is the energy cost that can meet the requirement of minimum power without path loss. Compared with $E_{u}A^{-1}\left(d_{s},f\right)$, the circuit energy consumption can be ignored because it is the same for each sensor node. The main focus of this paper is that the node selection contributes to the influence of network lifetime due to different energy consumption of each node.

## 3. Optimized Node Selection Scheme

In the traditional sensor nodes’ selection scheme, each sensor node is selected by the same probability, which doesn’t consider the accuracy of reconstruction results and only guarantees that the signal can be reconstructed. Based on the Bayesian estimation scheme, an optimized sensor nodes’ selection scheme can be driven by considering the corresponding MSE and the network lifetime.

### 3.1. Scheme for Minimizing MSE of Estimation

Let $\tilde{\Phi }=\Phi^{T}\Phi$ be a diagonal matrix and $\tilde{\Phi }\in R^{N\times N}$ denote the sensor nodes’ selection matrix. If the *i*th diagonal element is 1, it means that the *i*th sensor node is selected. If the *i*th diagonal element is 0, it means that the *i*th sensor node is not selected. It is well known that the MSE of Bayesian estimation is the trace of error covariance matrix. By setting the sensor nodes selection matrix $\tilde{\Phi }$ as a variable and minimizing the MSE, the following optimization problem can be obtained:

(19) $$\begin{array}{c} \begin{array}{cc} \min_{\tilde{\Phi }}\; & Trace{\left(\sigma^{-2}\Psi^{T}\tilde{\Phi }\Psi +\Lambda \right)}^{-1},\\ s.t.\; & {\tilde{\Phi }}_{ii}\in \left\{0,1\right\},i=1,2,\cdots ,N,\\ & Trace(\tilde{\Phi })=M. \end{array} \end{array}$$

The first constraint condition decides whether the node is selected or not. The second constraint condition means that the number of selected nodes is *M*. Notice that the proposed optimization problem is non-convex due to the non-convexity of the first constraint condition, and it is difficult to obtain the optimal solution of it. To overcome this difficulty, the constraint condition is relaxed:

(20) $$\begin{array}{cc} \min_{\tilde{\Phi }}\; & Trace{\left(\sigma^{-2}\Psi^{T}\tilde{\Phi }\Psi +\Lambda \right)}^{-1},\\ s.t.\; & 0\leq {\tilde{\Phi }}_{ii}\leq 1,i=1,2,\cdots ,N,\\ & Trace(\tilde{\Phi })=M. \end{array}$$

By introducing a slack variable Q and applying the Schur’s complement, the optimization problem can be rewritten as follows:

(21) $$\begin{array}{cc} \min_{\tilde{\Phi },Q}\; & Trace(Q),\\ s.t.\; & \left(\begin{array}{cc} \sigma^{-2}\Psi^{T}\tilde{\Phi }\Psi +\Lambda & I_{N}\\ I_{N} & Q \end{array}\right)⪰0,\\ & 0\leq {\tilde{\Phi }}_{ii}\leq 1,i=1,2,\cdots ,N,\\ & Trace(\tilde{\Phi })=M. \end{array}$$

According to convex optimization theory, the optimization problem above is a semidefinite programming problem. A CVX tool cabinet in Matlab (R2017a, The MathWorks Inc., Natick, MA, USA) can be used to solve it effectively. There may be some other methods to solve it more efficiently, but it is beyond the scope of this paper.

### 3.2. Scheme for Prolonging the Network Lifetime

The network collects the information by putting the sensor nodes under the water. According to the feature of underwater environment, the channel differences of the sensor nodes are large. For example, the node with weak channel condition needs to take more power to transmit the signal so that the sink node can obtain the complete signal. However, the energy of each node is fixed. If there is a node with no energy prematurely, the sensor nodes’ network will be invalid. This leads to a serious waste of resources. Therefore, it is significant to provide a new node selection scheme for prolonging the lifetime of the sensor network. Assume that the initial energy of each sensor node is $E_{0}$. Let $E_{ij}$ denote the *i*th sensor node’s energy in *j*th selection. Then, let $P_{ij}=\frac{E_{ij}}{\sum_{i=1}^{N}E_{ij}}$ denote the proportion of the *i*th sensor node’s energy with the total energy in *j*th selection. The scheme aims at selecting the sensor nodes that hold more energy. Therefore, the optimization problem with the energy constraint condition in *j*th selection can be rewritten as follows:

(22) $$\begin{array}{cc} \min_{\tilde{\Phi },Q}\; & Trace(Q),\\ s.t.\; & \left(\begin{array}{cc} \sigma^{-2}\Psi^{T}\tilde{\Phi }\Psi +\Lambda & I_{N}\\ I_{N} & Q \end{array}\right)⪰0,\\ & Trace(\tilde{\Phi }P_{j})\geq C,\\ & 0\leq {\tilde{\Phi }}_{ii}\leq 1,i=1,2,\cdots ,N,\\ & Trace(\tilde{\Phi })=M, \end{array}$$

where $P_{j}=diag{\left(P_{1j},P_{2j},\ldots ,P_{Nj}\right)}^{T}$ and *C* is a constant threshold. From the energy constraint condition, it can be seen that the sensor nodes with less energy are selected by a small probability. If the sensor nodes with less energy are selected, it is possible that the energy constraint condition is not satisfied because the proportion values are small. For the final solution matrix, the *M* sensor nodes corresponding to the largest values of diagonal element in the solution matrix are chosen. The detail can be found in Algorithm 2.

**Algorithm 2** Optimized node selection algorithm based on convex relaxation.
1:Initialize the number of selected nodes *M*, threshold *C*, the number of selection as j=0, and $P_{0}$.2:**while** The residual energy of each node is non-negative. **do**3: j=j+1.4: Solve the optimization problem (22) by using CVX tool cabinet in matlab.5: The *M* sensor nodes corresponding to the largest values of diagonal element in the solution matrix Φ are chosen for data transmission.6: The signal is reconstructed by using Algorithm 1 or orthogonal matching pursuit (OMP) algorithm.7: Calculate $P_{j}$ according to the optimized solution matrix Φ.8:
**end while**
9:Output the number of selection *j* and $P_{j}$.


## 4. Simulation Results and Discussion

In this section, simulation results are provided for comparing our proposed optimized sensor nodes selection scheme with conventional compressed sensing scheme [31]. In [31], each sensor node sends data randomly with a probability or the fusion center deciding the nodes to send data randomly. The selection of nodes with a random scheme doesn’t consider the state and correlation of each node. OMP is an efficient algorithm that is usually used to reconstruct a signal [36]. This algorithm is also compared with our proposed algorithm in the simulation. We consider a sink node in the center of a circular region with the radius r=500 m. The sensor nodes are uniformly distributed in this region. For decreasing the computation complexity, the number of the sensor nodes is set as 64. The minimum distance between sink node and sensor node is 50 m. The minimum distance among sensor nodes is 40 m. Each sensor node acquires information and transmits it to sink node. The sink node collects all the information and requires that the minimum received power is $P_{0}=50$ mW. The discrete Fourier basis is applied in the evaluation. Assume that the energy of each sensor node is the same and is set as 600 J. The carrier frequency is assumed as 10 kHz. The constant *C* is set as $\frac{M}{N}$. The simulation shows mainly the performance of the reconstruction error and the lifetime of the sensor network. The reconstruction error is defined as follows:

(23) $$\begin{array}{c} \begin{array}{c} R_{e}=\frac{∥x-\hat{x}{∥}_{2}}{{∥x∥}_{2}}, \end{array} \end{array}$$

where x is the original signal and $\hat{x}$ is the reconstructed signal. If the residual energy of one sensor node is full, the sensor network is regarded as invalid. The lifetime of the sensor network is defined as the times that the sensor network can collect the information until the first sensor node died.

**Figure 1:**
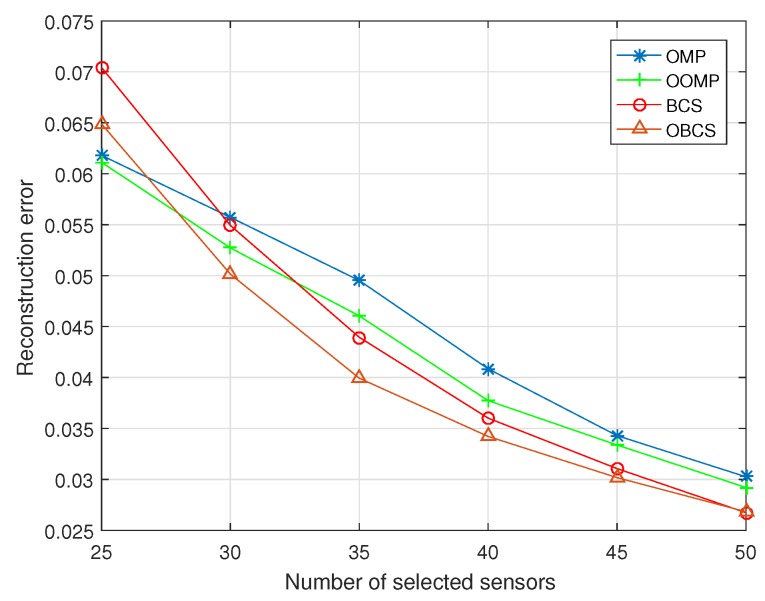
Reconstruction error versus different number of selected sensor nodes.

In Figure 1, the performance of reconstruction error is evaluated with the number of selected sensor nodes. It can be seen that the reconstruction error is decreasing when the number of selected sensor nodes increases. The proposed optimized sensor nodes selection scheme is superior to the conventional compressed sensing scheme. The performance of reconstruction scheme by using optimized Bayesian estimation outperforms the scheme with conventional Bayesian estimation. The reconstruction scheme by using optimized Bayesian estimation is better than the OMP algorithm and the optimized OMP algorithm when the number of selected sensor nodes is more than 30. When the number of selected sensor nodes is less than 30, the optimized OMP algorithm has better performance than the OMP algorithm and the optimized Bayesian estimation scheme. It is reasonable because the prior information is little when the number of selected sensor nodes is few. It is not suitable to use a Bayesian estimation scheme.

**Figure 2:**
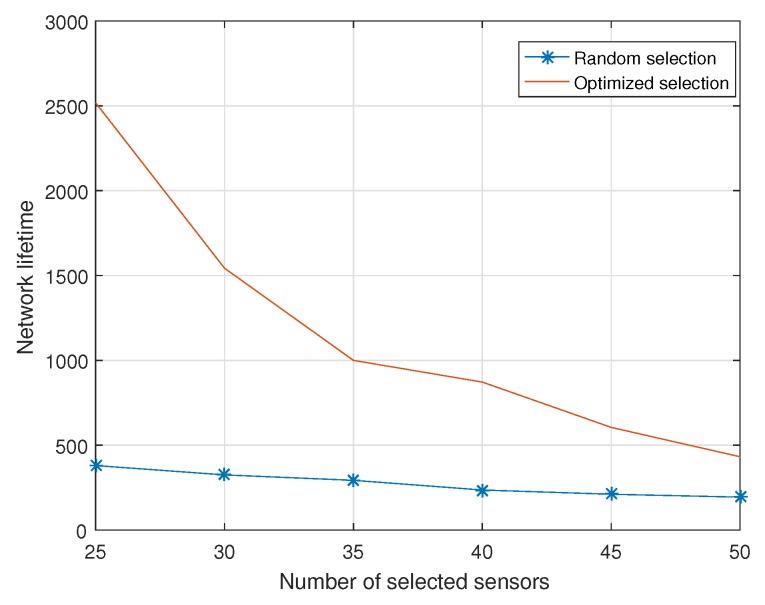
Network lifetime versus different number of selected sensor nodes.

Figure 2 shows the network lifetime versus the number of selected sensor nodes. When the number of selected sensor nodes grows, the network lifetime decreases. This is because the feasible selection schemes are less as the number of selected sensor nodes grows larger. It is obvious that the optimized sensor nodes selection scheme has a significant improvement than the conventional sensor nodes’ selection scheme. For example, when the number of selected sensor nodes is 25, the network lifetime with optimized sensor nodes selection scheme is 5.6 times more than conventional sensor nodes selection scheme.

This paper proposed an optimized sensor nodes selection scheme for compressed information acquisition based on Bayesian estimation. The proposed scheme is not limited to reconstructing the signal. In practice, the sink node can select different schemes to reconstruct the signal according to different performance requirements. For example, when the reconstructed signal is required to be accurate as much as possible, it is suitable to increase the number of selected sensor nodes and apply Bayesian estimation with an optimized selection scheme to reconstruct the signal. On the contrary, when the reconstruction error is not limited strictly, it is better to decrease the number of selected sensor nodes and use the OMP algorithm with an optimized selection scheme to reconstruct the signal for prolonging the network lifetime.

**Figure 3:**
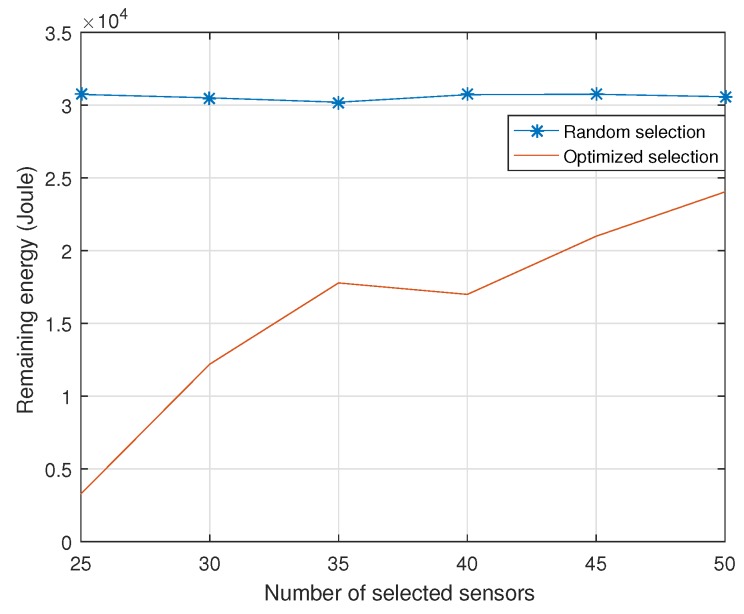
Remaining energy versus different number of selected sensor nodes.

Figure 3 shows the remaining energy of the sensor network versus the number of selected sensor nodes. It can be observed that the remaining energy of sensor network grows when the number of selected sensor nodes increases. We can see that the optimized sensor nodes’ selection scheme has more energy efficiency than the conventional sensor nodes’ selection scheme. For example, when the number of selected sensor nodes is 30, the optimized scheme makes full use of 2.3 times energy to prolong network lifetime more.

**Figure 4:**
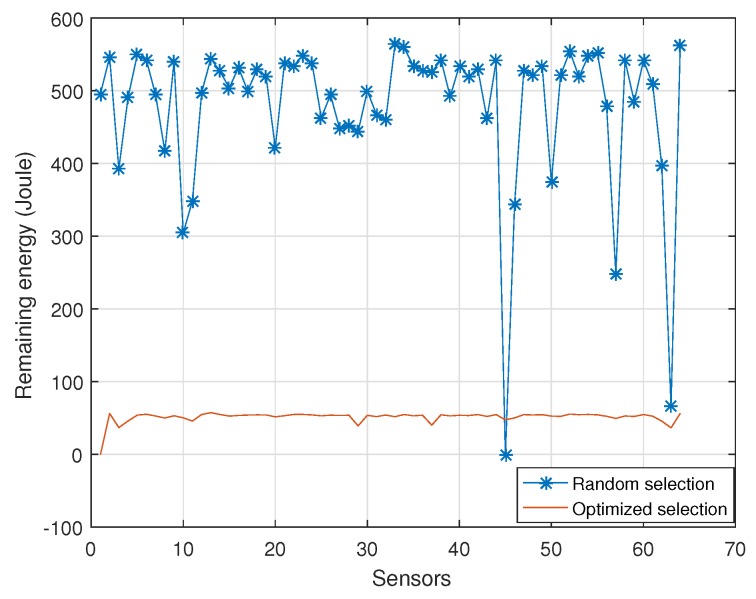
Remaining energy of each sensor node.

In Figure 4, the remaining energy of each sensor node is provided. The number of selected sensor nodes is set as 25. It can be seen that the remaining energy of each sensor is homogeneous for the optimized sensor nodes selection scheme. However, for the conventional sensor nodes selection scheme, the remaining energy of each sensor is disorderly. The remaining energy of some sensor nodes is few, and it is adequate for many sensor nodes. Thus, the use of random node selection scheme leads to the wasting of total energy.

**Figure 5:**
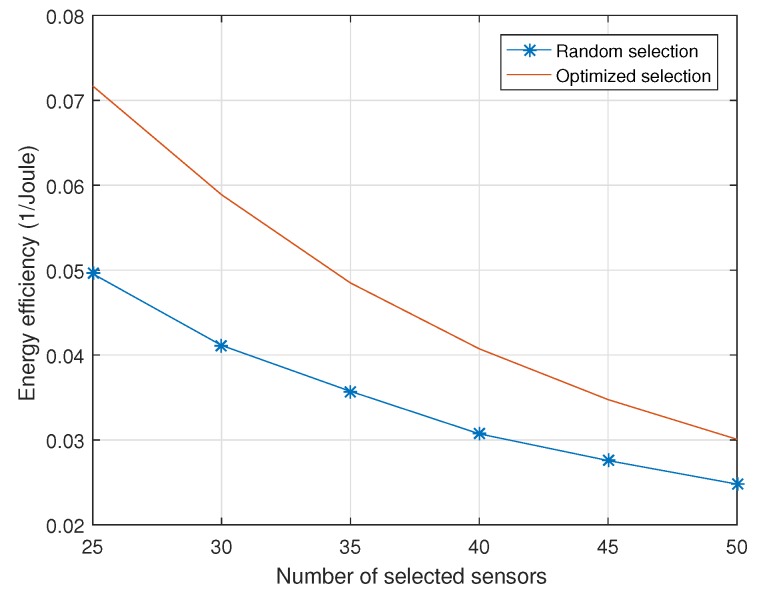
Energy efficiency versus different number of selected sensor nodes.

Figure 5 shows the energy efficiency versus different number of selected sensor nodes. The definition of energy efficiency is the proportion of network lifetime and the total energy cost. It represents the average time of information acquisition per unit energy. When the number of selected sensor nodes increases, the energy efficiency is decreasing because, as the number of selected sensor nodes grows larger, the energy cost in each information acquisition is also increased. The performance with the optimized sensor nodes selection scheme is better than the conventional sensor nodes selection scheme. By using the same energy cost, the proposed scheme can achieve more information acquisition times compared with the traditional scheme. Therefore, it can prolong the network lifetime.

## 5. Conclusions

In this work, an optimized sensor node selection scheme for compressed information acquisition is provided for improving the accuracy of reconstruction and prolonging the network lifetime. The signal reconstruction is achieved based on Bayesian estimation theory. The MSE of estimation can be obtained by the given error covariance matrix. Then, the proposed optimization problem aims at minimizing it by taking the selection matrix as the variable. By relaxing the non-convex constraint, the proposed optimization problem can be solved effectively. To increase the sustainability of the network, the scheme selects the nodes with more energy by adding a bound constraint in the optimization problem. Compared with the conventional node selection scheme, simulation results demonstrate that the proposed scheme can achieve more accurate reconstruction and improve the network lifetime significantly.

## Abbreviations

The following abbreviations are used in this manuscript:

NP	Non-deterministic polynomial
CSI	Channel state information
MSE	Mean square error
EM	Expectation maximization
OMP	Orthogonal matching pursuit
OOMP	OMP algorithm with optimized sensor nodes selection scheme
BCS	Bayesian compression sensing estimation
OBCS	Bayesian compression sensing estimation with optimized sensor nodes selection scheme

## Appendix A

To achieve the final sparsity prior, hierarchical priors about the hyperparameters α, $\sigma^{2}$ and the sparse vector s are given in this section. The hyperprior of α is defined as a Gamma prior distribution:

(A1) $$\begin{array}{c} \begin{array}{cc} p(\alpha ) & =\prod_{i=1}^{N}\Gamma \left(\alpha_{i}|a,b\right)\\ & =\prod_{i=1}^{N}\int_{0}^{+\infty }x^{a-1}e^{-x}dxb^{a}\alpha^{a-1}e^{-b\alpha }. \end{array} \end{array}$$

It can be seen that the prior information is not sufficient because the prior distribution of the variance $\sigma^{2}$ is unknown. The hyperprior of $\sigma^{-2}$ as a Gamma prior distribution is defined as follows:

(A2) $$\begin{array}{c} \begin{array}{cc} p(\sigma^{-2}) & =\prod_{i=1}^{N}\Gamma \left(\sigma^{-2}|c,d\right). \end{array} \end{array}$$

If a=b=c=d=0 holds, the hyperprior distribution becomes uniform distribution. In this paper, this assumption is kept. It is convenient to discuss the estimation of hyperparameters.

The posterior distribution of the unknown parameters s, α, and $\sigma^{2}$ is given by Bayes’s rules

(A3) $$\begin{array}{c} \begin{array}{c} p\left(s,\alpha ,\sigma^{2}|y\right)=\frac{p\left(y|s,\alpha ,\sigma^{2}\right)p\left(s,\alpha ,\sigma^{2}\right)}{p(y)}. \end{array} \end{array}$$

Considering that it is difficult to calculate p(y), which is the integral of $p\left(y|s,\alpha ,\sigma^{2}\right)p\left(s,\alpha ,\sigma^{2}\right)$, we decompose the posterior $p(s,\alpha ,\sigma^{2}|y)$ as follows instead of directly calculating it:

(A4) $$\begin{array}{c} \begin{array}{c} p\left(s,\alpha ,\sigma^{2}|y\right)=p\left(s|\alpha ,\sigma^{2},y\right)p\left(\alpha ,\sigma^{2}|y\right). \end{array} \end{array}$$

Then, the posterior of the sparse vector s can be obtained:

(A5) $$\begin{array}{c} p\left(s|\alpha ,\sigma^{2},y\right)=\frac{p\left(y|s,\sigma^{2}\right)p\left(s|\alpha \right)}{p(y|\alpha ,\sigma^{2})}. \end{array}$$
